# A Randomized Open-Label Trial of Artesunate- Sulfadoxine-Pyrimethamine with or without Primaquine for Elimination of Sub-Microscopic *P. falciparum* Parasitaemia and Gametocyte Carriage in Eastern Sudan

**DOI:** 10.1371/journal.pone.0001311

**Published:** 2007-12-12

**Authors:** Badria El-Sayed, Salah-Eldin El-Zaki, Hamza Babiker, Nahla Gadalla, Tellal Ageep, Fathi Mansour, Omer Baraka, Paul Milligan, Ahmed Babiker

**Affiliations:** 1 Department of Epidemiology, Tropical Medicine Research Institute, National Centre for Research, Khartoum, Sudan; 2 Faculty of Medicine, Sultan Qaboos University, Muscat, Oman; 3 Faculty of Medicine, University of Khartoum, Khartoum, Sudan; 4 Department of Epidemiology and Population Health, London School of Hygiene and Tropical Medicine, London, United Kingdom; World Health Organization, Switzerland

## Abstract

**Background:**

In areas of seasonal malaria transmission, treatment of asymptomatic carriers of malaria parasites, whose parasitaemia persists at low densities throughout the dry season, could be a useful strategy for malaria control. We carried out a randomized trial to compare two drug regimens for clearance of parasitaemia in order to identify the optimum regimen for use in mass drug administration in the dry season.

**Methodology and Principal Findings:**

A two-arm open-label randomized controlled trial was conducted during the dry season in an area of distinct seasonal malaria in two villages in Gedarif State in eastern Sudan. Participants were asymptomatic adults and children aged over 6 months, with low-density P. falciparum infection detected by PCR. Participants were randomized to receive artesunate/sulfadoxine-pyrimethamine (AS+SP) combination for three days with or without a dose of primaquine (PQ) on the fourth day. Parasitaemia detected by PCR on days 3, 7 and 14 after the start of treatment and gametocytes detected by RT-PCR on days 7 and 14 were then recorded. 104 individuals who had low density parasitaemia at screening were randomized and treated during the dry season. On day 7, 8.3% were positive by PCR in the AS+SP+PQ group and 6.5% in the AS+SP group (risk difference 1.8%, 95%CI −10.3% to +13.8%). At enrolment, 12% (12/100) were carrying gametocytes. This was reduced to 6.4% and 4.4% by day 14 (Risk difference 1.9% (95%CI −9.3% to +13.2%) in AS+SP+PQ and AS+SP groups, respectively.

**Conclusion:**

Addition of primaquine to artemisinin combination treatment did not improve elimination of parasitaemia and prevention of gametocyte carriage in carriers with low-density parasitaemia in the dry season.

**Trial Registration:**

ClinicalTrials.gov NCT00330902

## Introduction

In malaria endemic countries of sub-Saharan Africa, the majority of *Plasmodium falciparum* infections are asymptomatic and remain untreated. In eastern Sudan people who become infected during the wet season may retain chronic sub-microscopic asymptomatic infections throughout the dry season [1]–[3]. The presence of gametocytes within these sub-microscopic infections has been demonstrated using sensitive gametocyte-specific RT-PCR [4]–[6]. In a cohort study of 38 individuals monitored monthly by RT-PCR, 40% were found to retain gametocytes throughout the dry season [7]. Treatment of carriers to clear parasitaemia during the dry season may reduce the source of infection available to mosquitoes that emerge at the start of the rainy season. This could contribute to malaria control strategy if high coverage with an effective therapy is achieved.

Artemisinin combination treatment is highly effective in eliminating asexual parasitaemia, the source of merozoites committed for gametocyte production [8]. Since gametocyte longevity is limited [9] such treatment may lead, in the absence of reinfection, to eventual elimination of gametocyte carriage [10], [11]. Three doses of artesunate were found to reduce gametocyte carriage after treatment in clinical malaria cases [12]. Artemisinin derivatives inhibit gametocyte development but are not effective against mature gametocytes. Therefore to achieve more rapid clearance of mosquito-infective stages from the blood stream an actively gametocytocidal drug may be required [13]. Primaquine has been used for half a century as a hypnozoitocidal drug against *Plasmodium vivax*, as a prophylactic against all malaria species, and as a gametocytocidal drug against *P. falciparum* [14], [15]. The World Health Organization has recommended, for some areas, addition of a single dose of primaquine to treatment regimens for *P. falciparum* malaria to reduce transmission [16].

Individuals with low gametocyte densities undetectable by microscopy may still be infectious to mosquitoes [17], [18]. The combination of PCR and RT-PCR techniques allows the detection of very low density gametocyte producing *P. falciparum* infections [4], which persist as asymptomatic infections during the dry season. The present study was undertaken to compare the efficacy of AS+SP and AS+SP+PQ in treating low-density sub-microscopic *P. falciparum* parasitaemia before the transmission season in an area of marked seasonal transmission in eastern Sudan in order to identify the optimum regimen for use in mass drug administration during the dry season.

## Methods

The protocol for this trial and supporting CONSORT checklist are available as supporting information; see Checklist S1 and Protocol S1.

### Participants

The study was carried out in two villages in eastern Sudan. Abunaja Juama located at about 18 km south west of Gedarif and Kanara at 7 km south of Gedarif.

The whole area is characterized by seasonal malaria with transmission confined to three months of the year, October – December. The main malaria parasite is *P. falciparum* and the main mosquito vector is *Anopheles arabiensis* [2], [19], [20]. The proposal, informed consent form and the project information sheet were reviewed and approved by the Ethical Committee of the Federal Ministry of Health, Sudan. Preliminary meetings were held with the community leaders to ask for their permission. All adults and children aged 6 months or above who were resident in the two villages were invited to participate and to be screened for the presence of asymptomatic *P. falciparum*. Signed informed consent forms were obtained from all participants and from the parents or guardians of children under 15 years. Exclusion criteria included pregnancy, history of allergy to sulpha drugs, fever or other signs or symptoms of illness but none of the persons screened had any of these conditions. After screening individuals were excluded if, on microscopic examination of a blood film, Plasmodium species other than falciparum were detected or the sample was negative by PCR.

### Interventions

Participants were randomized to receive a standard dose of AS and SP (AS+SP) over three days, or AS+SP over the first three days plus primaquine (PQ) administered on the fourth day.

### Objectives

We wanted to determine the efficacy of AS+SP compared to AS+SP+PQ in clearing sub-patent parasitaemia and gametocyte carriage in persons with asymptomatic sub-microscopic *P. falciparum* parasitaemia, in order to identify the optimum regimen for use in mass drug administration in the dry season.

### Outcomes

The outcomes were *P.falciparum* parasitaemia detected by PCR on days 3, 7 and 14 and presence of gametocytes detected by RT-PCR on days 7 and 14. For these endpoints, a sample by finger prick was obtained on days 0, 3, 7 and 14 on glass slides and on filter paper, and on days 7 and 14 venous blood was collected in K_3_EDTA vacutainers. Adverse events were recorded on days 1, 2, 3, 7 and 14 and packed Cell Volume was measured on days 0, 7 and 14. Parasite DNA was extracted by the Chelex-Resin method [21]. A nested PCR method was employed to detect the small sub-unit ribosomal RNA gene (ssrRNA) [22] as a sensitive screening method for sub-patent parasitaemia. For detection of gametocytes by reverse transcriptase PCR (RT-PCR), mRNA of *P. falciparum* gametocyte specific *pfS25* gene was amplified in a nested PCR to increase sensitivity of the detection [4]. Thin and thick blood smears were prepared from all samples and stained in Giemsa's stain for ten minutes. Films were examined by two experienced technicians. Slides were considered negative if no parasite stage was detected after completing 100 negative high power fields.

### Sample size

In a previous trial [23] 30% of subjects were gametocyte positive on day 7 after treatment with SP and 3 doses of artesunate. Assuming a similar rate, a trial with 60 subjects in each arm would have at least 90% power (using a significance level of 0.05) to detect a difference between treatments if gametocyte prevalence detected by RT-PCR after treatment is 30% or more on day 14 in the AS+SP arm and 5% or less in AS+SP+PQ, allowing for 10% loss to follow up.

### Randomization—Sequence generation and allocation concealment

A cross sectional survey was carried out in the middle of the dry season (June) 2004 to identify sub-patent parasite carriers. A finger prick sample was collected on a glass slide for microscopic diagnosis and on filter paper for molecular analysis. Persons who were positive by PCR in June and eligible were enrolled in the study and randomly allocated into one of the two treatment arms. The list of carriers was sorted according to village and age to ensure that the treatment groups were balanced with respect to these two variables. The random allocation of this ordered list into the treatment arms was then created using restricted randomization with a block size of 12 in Stata version 7 (Statacorp, Texas). In August, a clinical examination was performed; no participants were excluded due to illness or other exclusion criteria. Before administering the first treatment dose (day 0), a venous blood sample was obtained from each individual in K_3_EDTA vacutainer tubes (Becton Dickinson) for gametocyte detection by RT-PCR, and a drop of blood was collected on a glass slide for microscopic diagnosis and another drop on filter paper for PCR.

### Randomization—Implementation

Treatment was administered under medical supervision. SP was administered on day 0 as 25 mg/kg sulfadoxine/1.25 mg/kg pyrimethamine for children under 50 kg, while adults and children weighing 50 kg or more were given three tablets (each tablet contained 500 mg of sulfadoxine and 25 mg of pyrimethamine). Artesunate was given on days 0, 1 and 2 at a dose of 200 mg (two tablets at 100 mg) for adults and for children weighing 50 kg or more, while children under 50 kg received 4 mg/kg body weight. Participants randomized to receive primaquine were, in addition, given a single dose of 0.75-mg/kg body weight on day 3. After each treatment dose, patients were observed for one hour, if the drug was vomited during the first half hour a full dose was repeated, if vomiting occurred after one hour half a dose was repeated.

### Blinding

The trial was not blinded; however laboratory staff performing the PCR assays were unaware of the treatment allocation.

### Statistical methods

95% confidence intervals were calculated for the difference between the proportions with parasitaemia and gametocytaemia in the two groups on days 7 and 14, using the modified score method of Newcombe ([24], method 10). Packed cell volume was compared using analysis of covariance, adjusting for the baseline measurement. Stata version 9 (Statacorp, Texas) was used.

## Results

### Participant flow

Out of a total of 615 asymptomatic individuals screened by microscopy in June 2004 (the dry season) 12 were positive for malaria parasites (9 were *P. falciparum*, 2 were *P. vivax* and one was *P. ovale*). 114/615 (19%) had PCR detectable *P. falciparum* parasitaemia (including the 9 which were positive by microscopy) and were therefore eligible for enrolment into the trial. The three who were positive by microscopy for other *Plasmodium* species were PCR negative for *falciparum*.

**Figure 1:**
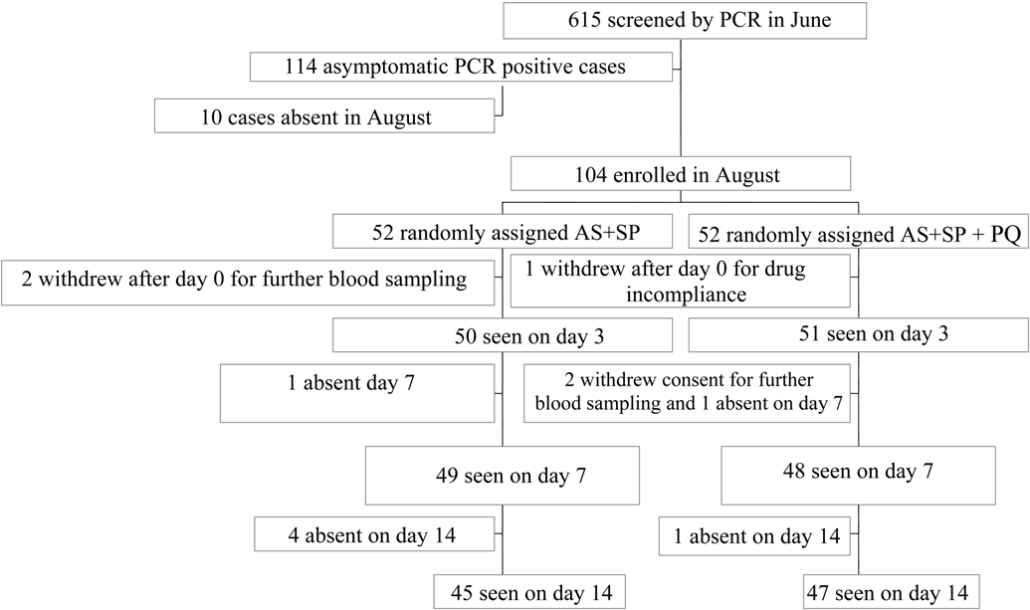
CONSORT Flowchart

The trial was started in August (the pre-transmission season) when 104 eligible participants were enrolled and 10 were absent. Of these 4 (4.2%) were positive for asymptomatic *P. falciparum* by microscopy. Five people withdrew consent; 2 in the AS+SP withdrew by day 3. While for the AS+SP+PQ group one withdrew on day 0 after the first dose and two by day 7 as shown in the trial profile Fig 1.

### Recruitment

Participants were enrolled from 17^th^ to 20^th^ August, and followed up until 3^rd^ September 2004.

### Baseline data

The demographic data for the study group is shown in Table 1.

**Table 1:** Characteristics of participants at enrolment.

	AS+SP (N = 52)	AS+SP+PQ (N = 52)
Age in years (mean, range)	18.9 (4–76)	24.8 (4–84)
Male: Female	22:30	23:29
*P.falciparum* detected by PCR	43 (83%)	52 (100%)
Gametocytes detected by RT-PCR	5/43 (10%)	6/52 (11.5%)
*P. Falciparum* detected by microscopy	0/52 (0%)	4/52 (8%)

### Numbers analyzed

Four (4%) individuals from those who were randomized in the AS+SP group were found to be negative by PCR on day 0. Of these 3 were lost to follow up on day 14 and one withdrew consent by day 3. They have been excluded from the analysis of safety and efficacy. The remaining withdrawn and lost to follow-up cases were also excluded. Data analysis was performed according to the protocol.

### Outcomes and estimation

On recruitment (day 0) 100 (96%) out of 104 individuals carried asymptomatic sub-patent *P. falciparum* infection. However, only 20 (20.4%) out of 98 participants seen on day 3 post-treatment were found to harbour *P. falciparum* infection detectable by PCR. On day 7 only seven (7.4%) out of 94 participants retained PCR detectable *P. falciparum* infection, 4/48 (8.3%) in the AS+SP+PQ group and 3/46 (6.5%) in the AS+SP group (risk difference 1.8%, 95%CI −10.3% to +13.8%). Similarly, five out of 92 seen on day 14 had PCR detectable parasitaemia (Table 2).

**Table 2:** Sub-patent parasitaemia and gametocyte carriage before and after Treatment.

Group		Day 0	Day 3	Day 7*	Day 14
AS+SP	Parasitaemia detected by PCR	84.6% (44/52)	25.5% (12/47)	6.5% (3/46)	4.4% (2/45)
	Gametocytaemia detected by RT-PCR	11.5% (6/52)	-	6.5% (3/46)	4.4% (2/45)
AS+SP+PQ	Parasitaemia detected by PCR	100% (52/52)	15.7% (8/51)	8.3% (4/48)	6.4% (3/47)
	Gametocytaemia detected by RT-PCR	11.5% (6/52)	-	8.3% (4/48)	6.4% (3/47)

* Risk difference between the two treatment groups on day 7: 1.9% (95%CI −8.5% to +12.3%), on day 14: 2% (95%CI −7.2% to +11.2%).

With regard to gametocytes, on enrolment, twelve (12%) out of 100 individuals carried gametocytes detectable by RT-PCR. None of them were gametocyte positive on day 7 or day 14. The same persons who were detected positive by PCR on days 7 and 14 were also positive for gametocytes by RT-PCR. On day 7, four (8.3%) out of 48 on AS+SP+PQ and three (6.5%) out of 46 individuals in AS+SP were found to have RT-PCR detected gametocytes (risk difference 1.9%, 95% CI: −8.5% to 12.3%). Similarly on day 14, 6.4% in AS+SP+PQ and 4.4% in AS+SP had sub-microscopy gametocytaemia (risk difference 1.9% (95%CI −9.3% to +13.2%).

Two out of the seven gametocyte carriers detected on day 7 retained their gametocyte producing infection until day 14. However, gametocytes reappeared on day 14 among three individuals who were gametocyte negative on day 7.

Packed cell volume was similar in both groups on day 7, mean 34.6% (15–44%) in the AS+SP group and 34.2% (26–42%) in the AS+SP+PQ group, difference between groups adjusted for baseline 0.78 (−0.75,2.3) P = 0.315. Similar results were seen on day 14 (Table 3).

**Table 3:** Mean Packed Cell Volume in the two treatment groups.

Day of follow up	SP+AS % (N)	SP+AS+PQ % (N)	Difference*
Day 0	35.7 (42)	36.1 (47)	
Day 7	34.7 (39)	34.2 (40)	0.78 (−0.75,+2.3) P = 0.32
Day 14	35.4 (34)	35.2 (41)	0.86 (−0.31,+2.0) P = 0.15

* Differences were estimated from regression, with the day 0 included as a covariant.

### Adverse events

No serious or severe adverse events were reported. Four of the participants (3.8%) vomited after the first treatment dose of AS+SP; 1 on AS+SP and 3 on AS+SP+PQ arm. One of these vomited immediately, refused to take the drug again and withdrew consent. The other three cases (2.9%) vomited after more than eight hours after taking the drug and the dose was therefore, not repeated for them. Two people complained of insomnia and another two complained of itching.

## Discussion

### Interpretation

We compared the efficacy of AS+SP and AS+SP+PQ in clearing asymptomatic sub-patent *P. falciparum* parasitaemia that persist in the dry season, as a potential control strategy in areas of seasonal transmission. After treatment with AS+SP alone 80% (78/98) of subjects with asymptomatic sub-patent *P. falciparum* infections became PCR negative by day 3 (before administration of PQ). However, only three (6.4%) in AS+SP and four (8.3%) in AS+SP+PQ group had sub-patent gametocytes on day 7, and two (4.4%) and three (6.4%) on day 14, respectively.

The present results are consistent with previous findings that asymptomatic sub-patent parasitaemia [1]–[3] and gametocytes carriage [6], [7] persist throughout the dry season among inhabitants of villages in eastern Sudan. The low prevalence of gametocytes compared to asexual stages has been reported previously. The ratio of gametocyte to asexual stages in *P. falciparum* was found to be less than 1∶10 [25]–[27], a recent study calculates a much lower ratio (1∶156) [28]. The positive correlation found between gametocyte density in the blood and infectiousness to mosquitoes [29]–[32] is considered to be controversial. It may be hampered by the low sensitivity of microscopy [33] and by transmission blocking immunity [34]. *P. falciparum* gametocyte carriers in nature usually harbour less than 100 gametocyte µl^−1^ blood and there is evidence that 1–10 gametocytes µl^−1^ blood are infectious to mosquitoes [17]. Therefore, the dry season sub-patent carriers represent an infectious source of *P. falciparum* to *Anopheles* mosquitoes [35] upon their reappearance during the rainy season. This has recently been demonstrated by a mosquito infectivity study in Kenya, which revealed high contribution of inhabitants with sub-patent *P. falciparum* infection to infectivity of *Anopheles* mosquitoes [35].

Our results show the efficacy of artesunate against asymptomatic sub-patent gametocyte carriage. Gametocytes present before treatment were most probably mature since we used the *PfS25* gene, which is exclusively expressed by mature gametocytes [36], [37]. Gametocyte sequestration could be the main reason for detection of some RT-PCR positive samples on days 7 and 14 [9]. It has been suggested that SP can release gametocytes from sites of sequestration, a process that can increase gametocyte density on day 7 to 14 post-treatment [38], but this was not borne out by results from a randomized trial in asymptomatic carriers [11]. However these gametocytes are not expected to be a major source for malaria transmission as, in absence of asexual forms, may live for an average of about 6.4 days [27]. This assumption is supported by the fact that five out of seven participants who were gametocyte positive on day 7 became negative by day 14. In spite of the safety of primaquine reported in this study, the low frequency of gametocytes did not allow us to demonstrate its importance conclusively but our results suggest that AS+SP without primaquine is an adequate regimen to clear the pre-transmission season gametocyte reservoir, which plays a central role in secondary cases arising at the start of the transmission season. Control strategies which target gametocyte carriage during the dry season could have strong impact on malaria morbidity and mortality in this area.

### Generalizability

The clearance of sub-patent parasitaemia and gametocyte carriage by day 7 after treatment with AS+SP indicates that this may be an effective treatment for use to clear low density *P.falciparum* parasitaemia in the dry season without the need for the addition of primaquine. These results, for infections near the limit of detection, may not apply to higher density infections where detectable gametocytes may persist for several weeks in spite of effective treatment of asexual parasitemia [38], although gametocytes will disappear over time when the asexual population has been eliminated. A limitation of the study is the sensitivity of the PCR that was used. PCR negative individuals may test positive if more sensitive methods are used and may still be able to infect mosquitoes. Another limitation is that the power to detect an effect of PQ on gametocyte carriage was limited.

### Overall evidence

The combination AS+SP without primaquine is effective in eliminating the dry season sub-microscopic parasitaemia and gametocyte carriage. Therefore, this regimen could be recommended for use in mass drug administration in the dry season to control malaria in areas of seasonal transmission.

However, it may be necessary to complete the drug administration before the rainy season and resurgence of the mosquitoes so as to avoid transmission of mature gametocytes.

## Supporting Information

Protocol S1 Trial Protocol
(0.16 MB DOC)

Checklist S1 CONSORT Checklist
(0.04 MB DOC)

## References

[1] Roper C, Elhassan IM, Hviid L, Giha H, Richardson W (1996). Detection of Very Low Level Plasmodium falciparum Infections using the Nested Polymerase Chain Reaction and a Reassessment of the Epidemiology of Unstable Malaria in Sudan.. Am J Trop Med Hyg.

[2] Babiker HA, Abdel-Muhsin AM, Ranford-Cartwright LC, Satti G, Walliker D (1998). Characteristics of Plasmodium falciparum parasites that survive the lengthy dry season in eastern Sudan where malaria transmission is markedly seasonal.. Am J Trop Med Hyg.

[3] Babiker HA, Abdel-Muhsin AA, Hamad A, Mackinnon MJ, Hill WG (2000). Population dynamics of Plasmodium falciparum in an unstable malaria area of eastern Sudan.. Parasitology.

[4] Babiker HA, Abdel-Wahab A, Ahmed S, Suleiman S, Ranford-Cartwright L (1999). Detection of low level Plasmodium falciparum gametocytes using reverse transcriptase polymerase chain reaction.. Mol Biochem Parasitol.

[5] Menegon M, Severini C, Sannella A, Paglia MG, Sangare D (2000). Genotyping of Plasmodium falciparum gametocytes by reverse transcriptase polymerase chain reaction.. Mol Biochem Parasitol.

[6] Abdel-Wahab A, Abdel-Muhsin AM, Ali E, Suleiman S, Ahmed S (2002). Dynamics of gametocytes among Plasmodium falciparum clones in natural infections in an area of highly seasonal transmission.. J Infect Dis.

[7] Nassir E, Abdel-Muhsin AM, Suliaman S, Kenyon F, Kheir A (2005). Impact of genetic complexity on longevity and gametocytogenesis of Plasmodium falciparum during the dry and transmission-free season of eastern Sudan.. Int J Parasitol.

[8] Talman AM, Domarle O, McKenzie FE, Ariey F, Robert V (2004). Gametocytogenesis: the puberty of Plasmodium falciparum.. Malar J.

[9] Eichner M, Diebner HH, Molineaux L, Collins WE, Jeffery GM (2001). Genesis, sequestration and survival of Plasmodium falciparum gametocytes: parameter estimates from fitting a model to malariatherapy data.. Trans R Soc Trop Med Hyg.

[10] Suputtamongkol Y, Chindarat S, Silpasakorn S, Chaikachonpatd S, Lim K (2003). The efficacy of combined mefloquine-artesunate versus mefloquine-primaquine on subsequent development of Plasmodium falciparum gametocytemia.. Am J Trop Med Hyg.

[11] Dunyo S, Milligan P, Edwards T, Sutherland C, Targett G (2006). Gametocytaemia after drug treatment of asymptomatic Plasmodium falciparum.. PLoS Clin Trials.

[12] Adjuik M, Babiker A, Garner P, Olliaro P, Taylor W (2004). Artesunate combinations for treatment of malaria: meta-analysis.. Lancet.

[13] Pukrittayakamee S, Chotivanich K, Chantra A, Clemens R, Looareesuwan S (2004). Activities of artesunate and primaquine against asexual- and sexual-stage parasites in falciparum malaria.. Antimicrob Agents Chemother.

[14] Bunnag D, Harinasuta T, Pinichpongse S, Suntharasami P (1980). Effect of primaquine on gametocytes of Plasmodium falciparum in Thailand.. Lancet.

[15] Grewal RS (1981). Pharmacology of 8-aminoquinolines.. Bull World Health Organ.

[16] WHO (1994). Antimalarial drug policies: data requirements, treatment of uncomplicated malaria and management of malaria in pregnancy.

[17] Muirhead-Thomson RC (1954). Factors determining the true reservoir of infection of Plasmodium falciparum and Wuchereria bancrofti in a West African village.. Trans R Soc Trop Med Hyg.

[18] Carter R, G, RW, J K (1980). Infectiousness and gamete immunization malaria.

[19] Hamad AA, Nugud Ael H, Arnot DE, Giha HA, Abdel-Muhsin AM (2002). A marked seasonality of malaria transmission in two rural sites in eastern Sudan.. Acta Trop.

[20] Bayoumi RA, Babiker HA, Ibrahim SM, Ghalib HW, Saeed BO (1989). Chloroquine-resistant Plasmodium falciparum in eastern Sudan.. Acta Trop.

[21] Plowe CV, Djimde A, Bouare M, Doumbo O, Wellems TE (1995). Pyrimethamine and proguanil resistance-conferring mutations in Plasmodium falciparum dihydrofolate reductase: polymerase chain reaction methods for surveillance in Africa.. Am J Trop Med Hyg.

[22] Snounou G, Viriyakosol S, Jarra W, Thaithong S, Brown KN (1993). Identification of the four human malaria parasite species in field samples by the polymerase chain reaction and detection of a high prevalence of mixed infections.. Mol Biochem Parasitol.

[23] von Seidlein L, Milligan P, Pinder M, Bojang K, Anyalebechi C (2000). Efficacy of artesunate plus pyrimethamine-sulphadoxine for uncomplicated malaria in Gambian children: a double-blind, randomised, controlled trial.. Lancet.

[24] Newcombe RG (1998). Interval estimation for the difference between independent proportions: comparison of eleven methods.. Stat Med.

[25] Kitchen SF, P P (1942). Observations on the mechanism of the parasite cycle in falciparum malaria.. Am J Trop Med.

[26] Dearsly AL, Sinden RE, Self IA (1990). Sexual development in malarial parasites: gametocyte production, fertility and infectivity to the mosquito vector.. Parasitology.

[27] Carter R, Graves P, McGregor WHWaIS (1989). Gametocytes.. Malaria: Principles and Practice of Malariology.

[28] Dyer M, Day KP (2003). Regulation of the rate of asexual growth and commitment to sexual development by diffusible factors from in vitro cultures of Plasmodium falciparum.. Am J Trop Med Hyg.

[29] Taylor LH, Read AF (1997). Why so few transmission stages? Reproductive restraint by malaria parasites.. Parasitol Today.

[30] Robert V, Molez JF, Trape JF (1996). Short report: gametocytes, chloroquine pressure, and the relative parasite survival advantage of resistant strains of falciparum malaria in west Africa.. Am J Trop Med Hyg.

[31] Graves PM, Carter R, McNeill KM (1984). Gametocyte production in cloned lines of Plasmodium falciparum.. Am J Trop Med Hyg.

[32] Sattabongkot J, Maneechai N, Rosenberg R (1991). Plasmodium vivax: gametocyte infectivity of naturally infected Thai adults.. Parasitology.

[33] Dowling MA, Shute GT (1966). A comparative study of thick and thin blood films in the diagnosis of scanty malaria parasitaemia.. Bull World Health Organ.

[34] Sauerwein RW, Eling WM (2002). Sexual and sporogonic stage antigens.. Chem Immunol.

[35] Schneider P, Bousema JT, Gouagna LC, Otieno S, van de Vegte-Bolmer M (2007). Submicroscopic Plasmodium falciparum gametocyte densities frequently result in mosquito infection.. Am J Trop Med Hyg.

[36] Kaslow DC, Quakyi IA, Keister DB (1989). Minimal variation in a vaccine candidate from the sexual stage of Plasmodium falciparum.. Mol Biochem Parasitol.

[37] Carter R, Gwadz RW (1980). Infectiousness and gamete immunization malaria.. Research in Malaria.

[38] Targett G, Drakeley C, Jawara M, von Seidlein L, Coleman R (2001). Artesunate reduces but does not prevent posttreatment transmission of Plasmodium falciparum to Anopheles gambiae.. J Infect Dis.

