# Supplementary material for: A Randomized Open-Label Trial of Artesunate- Sulfadoxine-Pyrimethamine with or without Primaquine for Elimination of Sub-Microscopic P. falciparum Parasitaemia and Gametocyte Carriage in Eastern Sudan
Source: PLoS One. 2007 Dec 12;2(12):e1311. doi: 10.1371/journal.pone.0001311 (PMC2111052; doi:10.1371/journal.pone.0001311)
Supplement: Protocol S1 — Trial Protocol (0.16 MB DOC) [file pone.0001311.s001.doc]

**WORLD HEALTH ORGANIZATION**

**ORGANISATION MONDIALE DE LA SANTE**

| **FOR OFFICIAL USE ONLY** | | |  | |  | | | |
| --- | --- | --- | --- | --- | --- | --- | --- | --- |
| **Date of receipt** | | Research area | | | | **Serial number**  **SGS03/……..** | | |
| RESEARCH PRE-PROPOSAL FORM | | | | | | | | |
| **1. Name of the Principal Investigator** | | | | | | | | |
| Last name: El Sayed | First name(s) Badria Babiker | | | | | | | Sex: F |
| Title: Doctor |  | | | | | | | |
| Occupation | Head of Epidemiology Department | | | | | | | |
|  |  | | | | | | | |
| Full postal address of the Principal Investigator for official communication | (Office)  Tropical Medicine Research Institute, P. O. Box 1304, Khartoum, Sudan  (Home) | | | | | | | |
|  | Tel (o): + 249 11 779246 | | | Tel (h): + 249 15 593650 | | | | |
|  | Fax: + 249 11 781845 | | | e-mail (mandatory): badriab@hotmail.com | | | | |
| **2. Name of other investigators** | | | | | | | | |
| Family name: Malik | First name(s) Elfatih Mohamed | | | | | | Sex: M | |
| Title: Doctor | | | | | | | | |
| Occupation | Assistant professor of Community Medicine | | | | | | | |
|  |  | | | | | | | |
|  |  | | | | | |  | |
|  | | | | | | | | |
|  |  | | | | | | | |
|  |  | | | | | | | |
| **3. Title of Project** (120 characters maximum, clearly covering the entire study)  Antimalarial drug combinations for controlling the dry season sub-patent parasitaemia and *P. falciparum* gametocyte reservoir in an area of seasonal transmission, in Sudan. | | | | | | | | |

| **4. Background:**  (Literature review of previous studies on the subject; Justification of the study by stating the problem and its public health significance)  Based on mathematical modelling Macdonald^1^ suggested that malaria eradication could be achieved by repeated mass administrations with at least 80% coverage with an effective drug, and this was most likely to be successful in areas of seasonal transmission where the basic reproduction rate of the infection is low (below 4 or 5). It was recommended that mass drug administration (MDA) should be administered repeatedly during the dry season, in combination with anti-vectorial measures that would also be delivered to most effect during the dry season. MacDonald’s approach was used in 1966-1968 in the Kankiya District in Northern Nigeria with chloroquine + pyrimethamine in combination with DDT house spraying^2^. The intervention reduced asexual and gametocyte parasite rates to very low levels during the dry season but did not interrupt malaria transmission and malaria prevalence during the peak of each transmission season. This was attributed in part to a failure of the insecticide campaign, the comparability of the control area and the predictions of the mathematical model. The Garki Project conducted from 1970 to 1975 in Kano State, Nigeria, achieved a high level of control but failed to interrupt transmission^3^. The trial included treatment with sulfalene-pyrimethamine during the dry season with a coverage of 85%, and residual spraying. The failure was attributed primarily to incomplete coverage of MDA and to the persisting high vectorial capacity. Malaria control using insecticide impregnated bednets, larviporous fish and multiple rounds of MDA succeeded in eliminating malaria from Aneityum, Vanuatu^4^. In 1998 in The Gambia, the effect of mass administration of SP plus one dose of artesunate at the end of the dry season in reducing malaria incidence was evaluated using a community randomized trial design^5^. The trial showed a transient reduction in incidence in the intervention villages, which may be partly or wholly attributable to a persisting direct protection from SP, but there was no overall benefit of MDA in terms of the total incidence over the whole surveillance period. The failure to achieve a more lasting reduction in incidence was attributed to persistence of mature gametocytes into the period of expansion of mosquito numbers, and the high vectorial capacity.  The previous studies described have three important limitations. Firstly, the drugs and drug regimens used have limited direct gametocidal action, their gametocidal effect is primarily indirect, through elimination of asexual parasites, and some of them have little effect on developing gametocytes. There would therefore be only a gradual reduction in infectivity after drug treatment, but during this time there is a risk of re-infection. Macdonald assumed treatment immediately clears gametocytaemia. The second limitation has been the use of microscopy for gametocyte detection, specially that patients who acquired infections during the wet season were found to harbor sub-patent gametocytemia through the following dry season in areas of seasonal transmission^6^. Individuals with gametocyte densities too low to detect by conventional methods may still be infectious to mosquitoes^7, 8^. The third limitation is the selection of an appropriate drug and the evaluation of its effects.  A multiple dose regimen delivered earlier in the dry season, or intermittently throughout the dry season, may have been more effective in eliminating gametocytaemia before the onset of widespread transmission. If the drug has no direct effect against gametocytes, we expect to see an initial increase in the density of gametocytes following treatment of a malaria attack, reflecting the natural wave of gametocytaemia, and then a gradual decrease in density. This wave of gametocytaemia would not be expected after treating asymptomatic individuals, and this is borne out by a recent randomized trial in The Gambia where asymptomatic individuals positive for asexual P.falciparum were randomized to receive treatment with placebo, SP, or SP combined with a single dose of artesunate. Gametocyte prevalence did not increase after any of the treatments. Gametocyte prevalence decreased steadily in both the SP and SP+ART groups (S Dunyo, unpublished data).  Some drugs lead to more rapid decline in gametocytaemia than others but there is no substantial evidence of any drug having an immediate direct effect on circulating mature gametocytes. Gametocidal properties of antimalarial drugs are reviewed by Butcher^8^.  The most useful of the currently available data on drug effects on gametocytes comes from microscope detection in blood smears made at intervals after treating malaria patients. No work was done on testing the efficiency of antigametocidal drugs in clearing gametocytes from individuals who harbor sub-patent gametocytemia detected by more sensitive methods like the RT-PCR. |
| --- |
| **5. Objectives of the study:**  **5.1 General objective** (overall objective, one paragraph maximum)  To identify the appropriate drug which can be used for treating *P. falciparum* sub-patent parasitaemia and gametocyte reservoir during the dry season in an area of marked seasonal malaria transmission in eastern Sudan. The ultimate aim is to reduce malaria morbidity and mortality during the transmission season.  **5.2 Specific objectives:** (should cover the details of the general objective)   - To identify individuals with sub-patent parasitaemia during the dry season using nested PCR technique - To identify gametocyte carriers among them using RT-PCR - To carry out a community-based intervention study for testing the efficiency of two drug combinations; Artesunate plus Sulphadoxine Pyrimethamine (S/P) and Artesunate plus Sulphadoxine Pyrimethamine (S/P) plus primaquine in clearing sub-patent parasitaemia and gametocytaemia from carriers at the end of the dry season. - To evaluate the effect of such a strategy on malaria morbidity and mortality during the transmission season.   **5.3 Secondary objectives (optional):** these are subsidiary objectives that could be studied during the course of the project but are not the main objectives of the study.  None |
| **6. Materials and Methods** (that could best achieve the study objectives)  **6.1 Study area/setting** (description of the area or setting where the study will be conducted)  The study will be carried out in two villages in eastern Sudan. These villages have relatively stable conditions and no modifications in the applied control measures are expected. They are far from each other and matched for malaria burden, environmental factors and control measures.  The whole area is characterized by seasonal malaria transmission. The season is confined to three months of the year, October – December. The main malaria parasite is *P. falciparum* and the main mosquito vector is *Anopheles arabiensis*^10^. Subpatent parasitaemias persist in some individuals throughout the dry season, with re-appearance of clinical symptoms in the malaria season associated with reinfection^11^.  **6.2 Study subjects** (eligibility and exclusion criteria for the study subjects)  The whole residents of the two villages will be recruited into the study. The following ones will be excluded from drug testing :   - pregnant women - children < 3 year old - history of allergy to sulpha drugs - infection with *Plasmodium* species other than *falciparum*   6.3 Combination Therapy:  The antimalarial drug combinations would be: Artesunate (AS) + Sulphadoxine Pyrimethamine (S/P) + primaquine (single dose of 0.75 mg of base/kg body weight) for one group and AS + S/P for the other. These are intended to treat malaria cases, sub-patent parasitaemia and gametocyte carriage. AS and S/P are blood schizontocidal drugs active against *P. falciparum* and gametocytocidal against developing gametocytes of *P. falciparum*. The addition of premaquine is expected to clear mature gametocytes.    6.4 Study design  A community-based intervention study will be conducted in the two villages. The study will start by a baseline evaluation of malaria morbidity and mortality during the transmission season of 2003.  A cross-sectional clinical, parasitological and molecular survey will be carried out during the dry season of 2004. Villagers will be screened for malaria parasite by microscopy. Samples detected negatives by blood films will be screened by conventional nested PCR *Pfs25* primers for detection of sub-patent parasiteamia. Blood will be collected from individuals detected positive by PCR for detection of gametocyte using the RT-PCR technique.  A treatment trial will then be carried out on malaria cases and asymptomatic carriers at the end of the dry season to test the efficacy of two combinations of antimalarial drugs. They will be followed up on days 3, 7 and 14 to confirm the clearance of *P. falciparum*  sub-patent parasitaemia and gametocyte carriage.  The impact of treating sub-patent parasitaemia and gametocyte reservoir on malaria morbidity and mortality will then be evaluated by carrying out a post-intervention survey in the two villages during the next transmission season.  **6.5 Sample sizes**  **6.5.1 Sample size for detection of parasite positive individuals during the dry season:**  The prevalence of sub-patent parasitemia during the dry season is expected to be about 15%. Therefore, the whole two villages will be screened during the dry season for detection of all positives including microscopical cases and subpatent parasitaemia to give the sample size needed for treatment trial.  **6.5.2 Sample size for evaluation of the efficacy of the two combinations**  A trial with 55 subjects on each arm would have 90% power (using a significance level of 0.05) to detect a difference between treatment groups if gametocyte prevalence detected by RT-PCR after treatment is 20% or more on day 3 in AS/SP combination and 5% or less in AS/SP + PQ, allowing for 10% loss to follow up. From earlier surveys the prevalence of sub-patent parasitaemia in the dry season was expected to be about 15% therefore 650 residents of all age groups had to be screened |
|  |
| **6.5.3 Sample size for detection of the impact of treatment on malaria morbidity and mortality**  Based on the previous records the prevalence in the two villages is expected to be around 10%. Therefore, 216 residents from each village, out of the total of 500, will be examined to detect prevalence between 5 - 15 % at 5% level of significance with a design effect of 2.  The sample sizes were calculated using Epi-Info 2002 computer programme.  6.6. Sampling technique and randomization  Persons who were positive by PCR in the dry season will be enrolled in the study for measuring the efficacy of each combination. The list of eligible participants will be sorted according to village and age, to achieve an implicit stratification. The allocations for this ordered list will be generated using restricted randomization with a block size of 12 in Stata version 7 (Statacorp, Texas)  Simple random sampling technique will be used for the selection of the sample size needed for detecting the impact of the treatment on malaria morbidity.  6.7. Data Collection methods, instruments used, measurements  6.7.1.Describe the instruments used for data collection (questionnaire,observation recording form, etc..), studied variables included in these instruments, and methods used to test for the validity and reliability of the instrument.   - Clinical data will be collected by a medical doctor using a questionnaire. - Thin and thick blood films will be collected on slides by finger brick - A parallel drop of blood will be collected on filter paper from the same finger brick for screening by conventional nested PCR technique. - Microvet tubes will be used to collect 150 ul of blood by finger brick from PCR positive individuals only. Samples will be used for detection of subpatent gametocytes by RT-PCR.     6.7.2.Techniques used should be briefly described and referenced  Thin and thick blood films will be stained and used for species identification and parasite density estimation^13^.  **Detection of low level *P. falciparum* infection during the dry season**  *P. falciparum* DNA will be extracted from filter papers of microscopically negative blood samples^14^.  Nested PCR will then be used to detect low level *P. falciparum* infections that prevail during the dry season^15^. Principal genes to be detected are *Pfs*25 and *Pfg*377, which encode gametocyte specific proteins. Results will then be visualized using gel electrophoresis. Detection of gametocytes using RT-PCR RT-PCR method will be used for detection of sub-patent gametocytemia during the dry season^16, 17^. Briefly, blood samples collected for RNA isolation and RT-PCR of *Pfs25* and *Pfg377* will first be left at room temperature for 30 minutes, to allow exflagellation and microgamete formation. The plasma and the buffy coat will be removed and the red blood cells stored at -70°C. Total RNA will be isolated using RNA isolation kits. RT-PCR of RNA will then be carried out.   - - 1. **Study definitions (eg case definition) should be mentioned.**   Clinical malaria includes those with microscopically diagnosed *P. falciparum* and clinical symptoms.  Sub-patent parasitaemia includes those detected positive by PCR  Gametocyte reservoirs include those detected positive by the RT-PCR.  **6.8.Data Management and Statistical Analysis**  (Describe the overall plan and tests used for data analysis and statistical package used)  The efficiency of the two treatment regimens will be assessed by comparing the proportions detected positive for asymptomatic infections before and after treatment. Comparison will be done using chi-square test and the significance of the results will be measured by the *P-value*.  Malaria prevalence and percent of mortality will be determined from data collected during the transmission season survey. They will be used for evaluation of the intervention by comparing them between the two villages and with data of the previous transmission season for every village. Comparison will also be done by chi-square test.  The data will be analyzed using SPSS (Statistical Package for Social Science) version 10. |

| 1. Implications of study results on disease control    1. Expected results and potential contribution of the project to the relevant control programme   It is expected that by the end of this study the suitable and highly effective combination therapy of antimalarial drugs will be identified. The strategy of treating sub-patent parasiaetimia and gametocyte carriage during the dry season is expected to have great impact in malaria morbidity and mortality in the intervention villages during the transmission season. If the expected results are achieved, this strategy of treatment during the dry season with the identified drugs will be recommended to be incorporated into the national malaria control programme of the Ministry of Health for controlling malaria epidemics in areas of marked seasonal transmission.   - 1. Mechanisms to ensure implementation of research results in the health policy of the concerned control programme of the Ministry of Health - A community physician from Malaria, Schistosomiasis and Leishmaniasis Control programme, Federal Ministry of Health (Deputy Director), is co-investigator in the project. He will be in close contact with the progress of the project and its activities and will be aware by the results. - Copies of the progress reports and the final report will be sent to the Control Prgramme. - Formal meeting can be arranged with the staff of the control programme to discuss the findings of the project and the possibility of implementing them. |
| --- |
| 1. Areas of Integration of Research Activities (if applicable)   The project integrates very well with the activities of a national malaria project (SUD 06/21) receiving TC support from the International Atomic Energy Agency (IAEA). This project will benefit from its field and laboratory facilities. Dr Hamza Ali Babiker, U. of Edinburgh, will supervise the RT-PCR part and we will use the field station in Gadarief rent to his MRC malaria project in the area. Dr. Paul Milligan, Medical statistician in the MRC laboratories in the Gambia, greatly helped in the design and will help in statistical analysis and publication of the work. |
| 1. Bibliographic references (recent and relevant to the study subject and enumerated according to their order of appearance in the text)   1. Macdonald, G, Foll, CV and Cuellar, CB (1967) The potential value of mass treatment in malaria eradication. *WHO/MAL/67.615*.  2. Najera, JA, Shidrawi, GR, Storey, J, Lietaert, PEA (1973) Mass Drug Administration and DDT indoor-spraying as antimalarial measures in the Northern Savanna of Nigeria. *WHO/MAL/73.817*.  3. Molineaux L. & Gramiccia, G. ( 1980). The Garki project. Research on epidemiology and control of malaria in the Sudan Savanna of West Africa. *World Health Organization*, Geneva.  4. Kaneko A, Taleo G, Kalkoa M, Yamar S, Kobayakawa T, Bjorkman A. (2000). Malaria eradication on islands. *Lancet* **356**: 1560-1564.  5. von Seidlein, L, Walraven, GEL, Milligan, PJM et al. (2003). The effect of mass administration of pyrimethamine/sulfadoxine combined with artesunate on malaria transmission: a double blind, community randomised, placebo controlled trial in The Gambia. *Trans. R. Soc. Trop. Med. Hyg*. (in press).  6. Abdel-Wahab, A., Abdel-Muhsin, A. A., Ali, A., Sulaiman, S., Ahmed, S., Walliker, D., and Babiker, H. A. (2002). Dynamics of gametcytes among *Plasmodium falciparum* clones in natural infections in an area of highly seasona transmission. *Journal of Infectious Diseases*, **185**, 1838-1842.  7. Carter, R. And Gwadz, R. W. (1980). Infectiousness and gamete immunization malaria. Pages 263 – 297 in J. P. Kreier, ed., Malaria, Volume 3. Immunology and immunization. Academic Press, Inc., New York.  8. Muirhead-Thompson (1954) Factors determining the true reservoir of infection of Plasmodium falciparum and Wuchereria bancrofti in A west African village. *Trans. R. Soc. Trop. Med. Hyg*. **48(3)** 208-225.  9. Butcher, GA (1997) Antimalarial drugs and the mosquito transmission of Plasmodium. *International Journal for Parasitology* **27**: 975-987.  10. Bayoumi, R. L., Babiker, H. A., Ibrahem, S. M., Ghalib, H. W., Saeed,B.O., Khider, S., Elwasila, M., and Karim, E. A. (1989). Spread of chloroquine resistant Plasmodium falciparum in Sudan. *Acta Tropica* **46**: 157-165.  11. Babiker H.A., Bdel-Muhsin A.A, Ranford-Cartwright, L, Satti, G. & Walliker, D (1998) Characteristics of Plasmodium falciparum parasites that survive the lengthy dry season in eastern Sudan where malaria transmission is markedly seasonal. *Am. J. Trop. Med. Hyg* **59**: 582-590.  12. Bunnag, D., Harinasuta, T., Pinichpongse, S. And Suntharasamai, P. (1980). Effect of premaquine on gametocytes of *Plasmodium falciparum* in Thailand. *Lancet*, **2**: 91.  13. Bruce-Chwatt, L. J. (1993). Essential Malariology, 2nd edition by Gilles, H. M. and Warrell, D. A. The Bath  Press, U. K.  14. Plowe, C.V., Djimde, A., Bouare, M., Doumbo, O. & Wellems, T,E. (1995) Pyrimethamine & proguanil resistance-conferring mutations in P. falciparum dihydrofolate reductase: polymerase chain reaction methods for surveillance in Africa. *Am. J. Trop. Med. Hyg*., **52**: 565-568.  15. Snounou, G., Viriyakosol, S., Zhu, X., Jarra, W., Pinheiro, L., Rosario, V. E., Thaithong, S., Brown, K. N. (1993). High sensetivity of detection of human malaria parasites by the use of nested polymerase chain reaction. *Moleular and Biochemical Parasitology*, **61**, 315-320.  16. Babiker H.A., Abdel-Wahab A.O, Ahmed S., Suleiman S., Ranford-Cartwright, L, Carter R. & Walliker, D (1999). Detection of low level *Plasmodium falciparum* gametocytes using reverse transcriptase polymerase chain reaction (RT-PCR). *Mol. Biochem Parasitol*. **99**, 143-148.  17. Menegon M, Severini C., Sanella A, et al. (2000). Genotyping of *Plasmodium falciparum* gametocyte by RT-PCR. *Mol Biochem Parasitol*, **111**: 153 – 161. |
| 1. Ethical Considerations    1. Informed consent form (If needed, please attach extra documents)  - Preliminary meetings will be held with the community leaders to inform them about the project and its objectives and to ask for their permission and collaborating in passing the message to the community. - A signed informed consent form will be collected from householders stating their agreement to participate. Even within each household, blood samples will be collected from those who are willing to participate. - Blood samples will be collected by a qualified laboratory technician with experience of more than 15 years in this field. This will be done under supervision of a medical doctor. - Clinical investigations for all patients, including those with common diseases, during every survey will be carried out by a medical doctor. He will also be responsible for prescription of drugs and very close clinical monitoring specially for those who will be given antimalarial drugs. - The principal investigator is committed to provide drugs for malaria and for other common diseases to those who will be detected sick during the surveys.      - 1. Institutional ethical clearance   Do you have an ethical review board in your institution: Yes [ ] No [ X ]  If no, then the ethical clearance of the Ministry of Health could be accepted.  (To be amended in case of initial acceptance by the selection committtee) |
| 1. Other funding agency   Is your study funded by another funding agency: Yes [ x ] No [ X ]  (specify the agency and available funds)  TC grant from the IAEA. |

| **12. Timeframe of research activities** (please indicate the activities to be conducted and check the corresponding month) | | | | | | | | | | | | |
| --- | --- | --- | --- | --- | --- | --- | --- | --- | --- | --- | --- | --- |
| Activity | 1^st^ QUARTER | | | 2^nd^ QUARTER | | | 3^rd^ QUARTER | | | 4^th^ QUARTER | | |
|  | M1 | M2 | M3 | M4 | M5 | M6 | M7 | M8 | M9 | M10 | M11 | M12 |
| ***Transmission season of 2003 survey*** |  |  |  |  |  |  |  |  |  | **X** | **X** |  |
| ***Dry season field survey*** |  |  | **X** |  |  |  |  |  |  |  |  |  |
| ***Nested PCRs*** |  |  |  | *X* | *X* |  |  |  |  |  |  |  |
| ***Samples for RT-PCR*** |  |  |  |  | ***X*** |  |  |  |  |  |  |  |
| ***RT-PCRs*** |  |  |  |  | X | X |  |  |  |  |  |  |
| ***Submission of the Progress Report*** |  |  |  |  |  | X |  |  |  |  |  |  |
| ***Pre-transmission field survey*** |  |  |  |  |  |  | *X* |  |  |  |  |  |
| ***Treatment &***  ***Drug testing*** |  |  |  |  |  |  | X | X | X |  |  |  |
| ***Transmission season survey*** |  |  |  |  |  |  |  |  |  |  | ***X*** |  |
| ***Submission of the Final Report*** |  |  |  |  |  |  |  |  |  |  |  | X |

| **13. Outline of budget**    Total amount of budget: US$* 10 000 | |
| --- | --- |
| **Budget breakdown:** | **Budget (US$)** |
| Personnel**  _ Medical doctor  _ Two Technicians  _ Research assistant  _ Driver  The team will be involved in four field surveys, 5 days each | **2 000** |
| Supplies and Equipment  _ Reagents  _ Disposable  _ Chemicals  _ Consumables | **2 000** |
| Local Travel  To cover expenses of travel to the study area for four surveys (5 days each) and working there between the village and the field station in Gadarief town. | **3 500** |
| Patient Costs  Mainly for drugs | **1 500** |
| Training |  |
| Others (please, specify and justify briefly)  _  _  _  _ |  |
| **Total** | **9 000** |

*Not exceeding 10,000 US$

** Only a limited proportion of the funds should be allocated to the personnel

| **14. Other information (if needed, please add any other information)** |
| --- |
| **15. Annexes (Data collection instruments, elaboration on methods and procedures to be used, etc..) (Please attach the related documents)** |
